# Male Choice Hypothesis: Shortcomings, Inconsistencies, and Proposed Alternatives

**DOI:** 10.1007/s10508-026-03469-3

**Published:** 2026-07-13

**Authors:** Tobias Wilczkowski

**Affiliations:** Department of Psychology, Uppsala University, Box 1225, SE-751 42 Uppsala, Sweden

**Keywords:** Male choice hypothesis, Sexual fluidity, Female same-sex attractions, Bisexuality, Evolutionary psychology, Sexual orientation

## Abstract

The male choice hypothesis proposes that same-sex attractions among heterosexual women evolved primarily for male benefit, reducing the risk of extra-pair paternity and providing additional mating opportunities. However, this theory contains significant flaws. This article critically evaluates the male choice hypothesis, showing that it rests on assumptions about male fitness benefits that are inconsistent with established principles of evolutionary psychology and sexual selection and that it further suffers from internal contradictions and conflicts with the empirical evidence. Analysis shows that support for the hypothesis was inflated by a methodological error in which the unlabeled midpoint of their Likert-type scales was repeatedly treated as an affirmative response. Correction of this error in a reanalysis of the published data demonstrates that the authors’ own findings fail to support the hypothesis. Taken together, these issues demonstrate that the male choice hypothesis is not supported by either theoretical reasoning or the empirical evidence presented in its favor, and thus is unlikely to constitute a viable evolutionary explanation for same-sex attractions among heterosexual women. Finally, alternative evolutionary hypotheses are presented that provide more plausible explanations for same-sex attractions among heterosexual women.

**Supplementary Information:**

The online version contains supplementary material available at 10.1007/s10508-026-03469-3.

## Introduction

Same-sex sexual behavior is prevalent and unlikely to be entirely absent in any culture (Bailey et al., 2016), yet it presents a Darwinian paradox due to its apparent lack of direct reproductive or survival benefits (Monk et al., 2019), particularly in self-identified heterosexuals. The male choice hypothesis (Apostolou et al., 2017) proposes that heterosexual men evolved preferences for female partners who exhibit same-sex attractions or behavior, because such traits could confer reproductive advantages to men. While novel, this theory has substantial theoretical and empirical weaknesses. Several aspects conflict with established evolutionary principles, including sexual selection and parental investment, and contain internal inconsistencies that undermine its logical coherence. Additionally, Apostolou’s studies exhibit methodological issues, including misinterpretation of Likert scale midpoints and resulting distortions in reported findings, leading to overestimated support for the hypothesis. To address these concerns, all relevant data were reanalyzed using a corrected coding scheme, which demonstrates that the original findings fail to support the male choice hypothesis. In light of these shortcomings, it is important to consider alternative evolutionary explanations that more plausibly account for same-sex attractions in heterosexual women, situating this critique within a broader evolutionary framework.

## The Male Choice Hypothesis

### Proposed Function of Same-Sex Attractions in Heterosexual Women

The male choice hypothesis suggests that same-sex attractions in heterosexual women reduce cuckoldry risk for men, facilitate their access to other women, and may also strengthen the woman’s relationship with her partner.

#### Proposed Cuckoldry-Protection Mechanism

Throughout human evolution, men have faced the risk of cuckoldry—investing in offspring not genetically their own due to a partner’s infidelity—stemming from the fact that gestation occurs internally in the female body. This risk arises from extra-pair mating, but if a partner engages in same-sex contact instead of opposite-sex contact, cuckoldry risk is mitigated, as no conception occurs. The male choice hypothesis proposes that men evolved a preference for same-sex attractions in women as an adaptive strategy to counter this risk, selecting partners with this trait if expressed (Apostolou, 2020, p. 209; Apostolou et al., 2018, pp. 31–32). This preference is thought to be more advantageous in short-term relationships, where men have less control over their partner’s behavior and paternity certainty is lower, compared to long-term relationships (Apostolou, 2019a, p. 6; 2020, pp. 185, 187).

#### Proposed "Participation Effect" Mechanism

Beyond cuckoldry protection, a female partner’s involvement with other women can provide men the opportunity to participate in sexual activity with these third-party women. The male choice hypothesis posits that men whose female partners are attracted to women are more likely to gain additional partners, increasing reproductive success without acquiring a new mate, as the female partner facilitates this (Apostolou, 2020, p. 180; Apostolou & Christoforou, 2018b, p. 26). This advantage is thought to be greater in short-term relationships, where women are more likely to permit such involvement, unlike in long-term relationships where the risk of losing male investment is higher (Apostolou, 2020, p. 185; Apostolou et al., 2017, p. 374).

#### Proposed Mechanism for Enhanced Relationship Quality

The male choice hypothesis suggests that heterosexual women with same-sex attractions may experience enhanced relationship quality compared to those without this trait, as it is particularly desired by men, offering benefits like increased resources, attention, preferential treatment, and reduced punishment for infidelity (Apostolou, 2016b, p. 388; 2020, p. 208). This trait also allows women to redirect their partners’ extra-pair desires toward a third-party woman rather than an unrelated individual (Apostolou & Christoforou, 2018a, p. 140).

### Assumed Genetic Basis of Female Same-Sex Attractions

The male choice hypothesis posits that same-sex attraction in humans arises from genetic alterations, which may be inherited or influenced by environmental factors (Apostolou, 2016a, 2018a, 2020). According to this view, individuals typically carry alleles (alternative forms of a gene) that predispose them to other-sex attractions: Women are generally attracted to men and men to women. However, genetic alterations, including mutations, can result in alleles that predispose individuals to same-sex attractions. The more altered alleles that are present, the stronger the expression of same-sex attraction, shifting individuals along a spectrum: From exclusively heterosexual (with no same-sex attraction), to mostly heterosexual, to bisexual, to mostly or exclusively homosexual. Simultaneously, attraction to the opposite sex decreases. For example, if enough of these alleles are present, a woman may become exclusively attracted to women and not engage in opposite-sex mating. Over time, this reduction in opposite-sex mating could reduce reproductive success, potentially leading to the extinction of these alleles (Apostolou, 2020, p. 154; 2022a, p. 37). However, the persistence of same-sex attraction suggests this has not occurred. Historically, strong parental control over mate choice may have forced same-sex attracted women to marry men, maintaining reproduction and preventing allele extinction (Apostolou, 2007, 2017, 2020, 2023). Additionally, male preference for women with same-sex attraction has helped these alleles persist in the population (Apostolou, 2018a, 2020; Apostolou et al., 2017).

## The Male Choice Hypothesis: Theoretical and Empirical Limitations

### Structural Issues in the Theoretical Framework

The male choice hypothesis proposes that heterosexual men may select women with same-sex attractions for fitness benefits. However, this reasoning contains several logical inconsistencies.

If a man (Male 1) selects a woman (Female 1) with same-sex attractions, the hypothesis assumes her involvement with another woman (Female 2) would offer both cuckoldry protection and additional mating opportunities. Yet, heterosexual women with same-sex attractions are rare (Savin-Williams & Vrangalova, 2013), making such partnerships uncommon. Moreover, for this to lead to additional mating opportunities, Female 2 would need to consent to sex with Male 1, despite risks such as unplanned pregnancy and the lack of male investment. This contradicts the supposed benefit of same-sex attractions for women—acquiring more resources—since Female 1 would now need to secure resources for both herself and Female 2, reducing her own access to Male 1’s attention and support. Additionally, since women frequently engage in infidelity with other men for resources (Buss & Schmitt, 1993; Greiling & Buss, 2000), it suggests that ancestral women, reasonably including those with same-sex attractions, struggled to support themselves and their offspring without male support. Thus, Apostolou’s argument that these mostly heterosexual women, unlike exclusively heterosexual women, had the evolved ability to acquire resources not only for themselves, but for other individuals as well, is further evolutionarily implausible.

As a related problem, even if such short-term mating opportunities arose, the lack of biparental care—a key factor in offspring survival (Buss & Schmitt, 1993; Conroy-Beam et al., 2015)—remains problematic. The hypothesis assumes that, under a cuckoldry-mitigation framework, men could gain fitness benefits by mating widely without investment. However, in a species with highly dependent offspring, such a strategy would reduce offspring survival and thus undermine the proposed fitness advantage.

Furthermore, if Female 2 conceived with Male 1 and then sought resources from another male partner, it would create a cuckoldry risk for him, turning the supposed benefit of cuckoldry protection into a liability. These flaws pose serious challenges to the male choice hypothesis.

### Inconsistencies with Evidence on Sexual Fluidity and Gender-Nonspecificity

Further examination of the male choice hypothesis reveals additional inconsistencies. It claims that, among heterosexual women, only those who are mostly heterosexual possess the capacity to engage in same-sex activity. Yet, studies show that many exclusively heterosexual women consider and engage in same-sex activity (Apostolou & Christoforou, 2018a; Chandra et al., 2013; Diamond, 2008), highlighting sexual fluidity. Sexual fluidity refers to the capacity for a person’s sexual attractions or behavior to shift depending on context, rather than being fixed (Diamond, 2008). For example, a woman typically sexually attracted to men may, in some situations, be sexually attracted to women. Yet, Apostolou (2020, p. 10) dismisses sexual-attraction fluidity as a “myth,” despite overwhelming evidence supporting its existence (e.g., Bailey, 2009; Baumeister, 2000; Blumstein & Schwartz, 1977; Diamond, 2000, 2008; Dickson et al., 2013; Epstein et al., 2023; Golden, 1996; Kanazawa, 2017; Ott et al., 2011; Peplau & Garnets, 2000; Ross et al., 2012; Vrangalova & Savin-Williams, 2012).

Similarly, it rejects the well-established concept of gender-nonspecific objective sexual arousal among exclusively heterosexual women, i.e., physiological responses—such as increased genital blood flow—to sexual stimuli featuring the nonpreferred sex (e.g., Chivers et al., 2004; Lippa, 2017; Rieger & Savin-Williams, 2012; Safron et al., 2020; Snowden et al., 2024; Tsujimura et al., 2009; Wright & Adams, 1994). Apostolou (2020, p. 22) argues that self-identified exclusively heterosexual women exhibiting such responses are untruthful, insisting that only mostly heterosexual women are capable of experience such awareness.

This stance is perplexing, as overwhelming evidence for sexual fluidity and gender-nonspecific arousal in exclusively heterosexual women directly contradicts the male choice hypothesis’ genetic assumptions.

### Overstated Parental Control Over Daughters’ Sexual Behavior

Further inconsistencies highlight the infeasibility of the male choice hypothesis. It overstates parental influence—where forced mate choice by parents dictates daughters’ partners—in the evolution of female mate choice. In contrast, evidence shows that female promiscuity, based on free mate choice, has deep evolutionary roots, driving adaptations in male anatomy, physiology, and behavior. Men’s larger testicular size likely evolved due to substantial sperm competition within promiscuous females, favoring larger ejaculates (Buss, 2024; Leivers et al., 2014). Similarly, greater penis size and glans shape may help displace previous males’ seminal fluid, increasing fertilization chances (Gallup & Burch, 2004; Gallup et al., 2003). Additionally, sexual jealousy and mate-guarding behaviors likely evolved to counter female promiscuity (Buss, 1988; Buss & Shackelford, 1997). Thus, Apostolou’s claim that parental control selected for women’s same-sex attractions is flawed. Rather, strong parental control over daughters’ sexual behavior—along with practices like genital mutilation, forced body concealment, and threats (Apostolou, 2013; Blake et al., 2018; Muteshi et al., 2016)—is more likely an evolutionarily novel phenomenon.

### Absence of a Theoretical Trade-Off Between Opposite-Sex and Same-Sex Behaviors

The male choice hypothesis claims same-sex attractions result from genetic alterations that impose a rigid compromise between engaging in same-sex and other-sex behavior, but this contradicts scientific evidence. Homosexual women do (freely) engage in sexual activity with men (Diamant et al., 1999; Diamond, 2008), and bisexual women report *more* male partners than heterosexual women (Kanazawa, 2017), with reproductive output similar to exclusively heterosexual women (Apostolou, 2022b). Additionally, as discussed earlier, exclusively heterosexual women engage sexually with other women, further challenging the notion of an inherent trade-off between same-sex and other-sex behavior.

### Limitations of the Hypothesized Genetic Architecture

There is a tendency to overemphasize genetics in explaining complex traits like sexual orientation (Carver et al., 2017; Dar-Nimrod & Heine, 2011), a bias reflected in Apostolou’s male choice hypothesis.

A study of 477,522 people from the UK, USA, and Sweden (Ganna et al., 2019) found five genetic markers for same-sex behavior, accounting for less than 1% of variation, meaning they cannot predict behavior. Genetics accounted for 8 to 25% of the differences, with the rest attributed to environmental factors. Moderate genetic similarities between men and women suggested shared, but not identical influences (Diamond, 2021; Mills, 2019).

The genetics of ever engaging in same-sex behavior differed from those influencing the proportion of same-sex partners, meaning no single genetic path determines sexual preferences (Diamond, 2021; Ganna et al., 2019). Furthermore, no genetic link was found between individuals with exclusively same-sex partners and those with fewer than one-third, suggesting a genetic spectrum from moderate bisexuality to exclusive same-sex attraction, but excluding mild bisexuality, which should include heterosexuals with same-sex attractions (Diamond, 2021; Ganna et al., 2019). These findings contradict the male choice hypothesis’ genetic claims about same-sex attractions, which constitute the foundation of the hypothesis.

### Summary of Conceptual Limitations

- The male choice hypothesis’ framework contains contradictions that violate foundational evolutionary principles.
- It ignores evidence of sexual fluidity and gender-nonspecific arousal in women, challenging assumptions of fixed attractions.
- It overstates parental control over women’s sexuality, conflicting with evidence for evolved female promiscuity.
- It wrongly asserts that same-sex attraction directly correlates with disinterest in opposite-sex partners, a view unsupported by empirical data.
- It relies excessively on genetics, adopting a rigid, deterministic perspective inconsistent with recent findings.

## Review of Apostolou’s Own Data in Studies Testing the Male Choice Hypothesis

The male choice hypothesis, which posits that same-sex attractions in heterosexual women protect against cuckoldry and offer men more copulatory opportunities, has been tested in several studies by Apostolou et al. Before reviewing the results, however, it is crucial to address the serious methodological oversights present in these studies.

### Interpretation Issues in the Use of Likert-Type Scales

In the studies, Apostolou frequently uses a five-point Likert scale with only two labeled anchors: 1 ("*strongly*/*totally disagree*" or "*very unlikely*") and 5 ("*strongly*/*totally agree*" or "*very likely*"). The intermediate categories (2–4), including the midpoint (3), were entirely unlabeled and therefore had no assigned valence. Despite this, the midpoint was repeatedly treated as expressing positive valence—"moderate agreement" or "moderate likelihood"—instead of the neutral valence that midpoints typically represent in Likert-type response formats (Chyung et al., 2017; Likert, 1932). This misinterpretation led to a coding scheme in which responses of 3, 4, and 5 were classified as "affirmative," while 1 and 2 were classified as "unaffirmative." This practice appears across multiple publications (Apostolou, 2016b, p. 385; Apostolou, 2019b, p. 4; Apostolou & Christoforou, 2018a, p. 137; Apostolou et al., 2017, p. 375; 2018, p. 34; Wang & Apostolou, 2019, p. 4). Treating an unlabeled midpoint as affirmative inflates apparent agreement with key items, producing results that appear to support the male choice hypothesis even when unweighted frequencies do not. At the same time, the same distortion biases other findings in the opposite direction, creating internal inconsistencies between results that seem to support and oppose the hypothesis. Because this coding practice systematically alters the valence of the data, the conclusions drawn from these studies cannot be interpreted at face value.

### Methods for Reanalysis

To correct for the scale misinterpretation described above, all relevant data were reanalyzed. The reanalysis presented in both the main text and the online supplement is based exclusively on the percentage distributions reported in tables across Apostolou et al.’s studies, primarily derived from Likert-type items but also including percentage-based categorical responses where applicable, as no raw datasets were available. Access to the original datasets would have been preferable, but because the relevant information is reported in the published tables, the analyses could be reproduced using the reported percentage distributions. Because the midpoint of the Likert-type scales carried no defined valence and the raw data were unavailable, it was not possible to conduct a midpoint-retained analysis without making unsupported assumptions about its meaning.

In this reanalysis, the unlabeled midpoint (3) was treated as neutral and therefore excluded. Scores of 1 (strongly/totally disagree) and 2 (disagree) were coded as unaffirmative, while scores of 4 (agree) and 5 (strongly/totally agree) were coded as affirmative. Results derived from this corrected coding are referred to as "unskewed results," while the original published results are referred to as "original results." When original results are presented, the corresponding unskewed values are shown for comparison.

For studies with differing sample sizes, weighted percentages were calculated. All statistical comparisons between skewed and unskewed proportions were conducted using two-sided two-sample proportion *t*-tests. Effect sizes were expressed using Cohen’s *h* for differences between proportions. Because multiple comparisons were conducted, the Benjamini–Hochberg procedure was applied to control the false discovery rate. Analyses were performed using Statistics Kingdom (2025).

### Findings on Women’s Self-Reported Same-Sex Preferences

A study of Greek-Cypriot samples examined heterosexual women’s attitudes and excitement toward extra-pair same-sex encounters within heterosexual relationships (Apostolou & Christoforou, 2018a). Unskewed results were extracted, and the authors’ own method for estimating appropriate weights was applied, considering the study’s focus on heterosexual women (pp. 137–138).

#### Same-Sex Contact Without Partner Participation

In response to their partner’s expressed desire for them to have sex with another woman, in long-term relationships, Apostolou’s original data showed that 31.3% found it sexually exciting, 24.7% would consider it, and 16.7% would do it. However, the unskewed data show that only 14.0% found it sexually exciting (while 64.7% did not, *p* < .001), only 12.2% would consider it (while 65.3% would not, *p* < .001), and only 5.8% would do it (while 73.3% would not, *p* < .001). In other words, the unskewed data show significant differences in the opposite direction to what Apostolou predicted.

In short-term relationships, Apostolou’s original data showed that 29.5% found it sexually exciting, 16.5% would consider it, and 12.6% would do it. However, the unskewed data show that only 15.3% found it sexually exciting (while 65.8% did not, *p* < .001), only 6.8% would consider it (while 73.1% would not, *p* < .001), and only 5.0% would do it (while 77.4% would not, *p* < .001) (Table 1). A two-sample proportion *t*-test based on the unskewed results revealed no significant differences between the long-term and short-term contexts in the propensity to find it sexually exciting (*z* = 0.70, *p* = .489) or to do it (*z* = 0.67, *p* = .506).^1^

**Table 1:** Women’s preferences for same-sex sexual contact

Same-sex Sexual Contact Without Partner Participation (Apostolou & Christoforou, 2018a)											
All Women – Long-Term Relationships											
		Original results (%)			Unskewed results (%)						
Reactions	*N*	1–2	3–5	*p*	1–2	4–5	*SE*	*z*	*p*	95% CI	*h*
I would do it	707	73.3	16.7	< .001	73.3	5.8	0.03	− 25.96	< .001	[− 0.71, − 0.64]	1.57
I would consider doing it	707	65.3	24.7	< .001	65.3	12.2	0.03	− 20.49	< .001	[− 0.57, − 0.49]	1.17
I would find it sexually exciting	707	64.7	31.3	< .001	64.7	14.0	0.03	− 19.51	< .001	[− 0.55, − 0.46]	1.10
I would be disgusted	707	42.4	47.2	**.070**	42.4	30.7	0.03	− 4.57	< .001	[− 0.17, − 0.07]	0.24
I would consider breaking up	707	42.4	47.6	**.049**	42.4	29.9	0.03	− 4.89	< .001	[− 0.17, − 0.08]	0.26
I would get angry	707	34.2	55.8	< .001	34.2	39.4	0.03	2.03	.043	[0.002, 0.10]	0.11
I would feel sad	707	30.1	59.9	< .001	30.1	42.9	0.03	5.00	< .001	[0.08, 0.18]	0.27
I would feel uncared for	707	34.9	55.1	< .001	34.9	36.3	0.03	0.55	**.583**	[− 0.04, 0.06]	0.03

All Women – Short-Term Relationships											
Reactions	*N*	1–2	3–5	*p*	1–2	4–5	*SE*	*z*	*p*	95% CI	*h*
I would do it	707	77.4	12.6	< .001	77.4	5.0	0.03	− 27.66	< .001	[− 0.76, − 0.69]	1.70
I would consider doing it	707	73.1	16.5	< .001	73.1	6.8	0.03	− 25.45	< .001	[− 0.70, − 0.63]	1.52
I would find it sexually exciting	707	65.8	29.5	< .001	65.8	15.3	0.03	− 19.34	< .001	[− 0.55, − 0.46]	1.09
I would be disgusted	707	44.0	46.0	**.450**	44.0	30.6	0.03	− 5.21	< .001	[− 0.18, − 0.08]	0.28
I would consider breaking up	707	28.0	62.0	< .001	28.0	43.8	0.03	6.19	< .001	[0.11, 0.21]	0.33
I would get angry	707	38.6	51.4	< .001	38.6	33.8	0.03	− 1.88	**.060**	[− 0.10, 0.002]	0.10
I would feel sad	707	47.3	42.7	< .001	47.3	24.7	0.03	− 8.85	< .001	[− 0.27, − 0.18]	0.48
I would feel uncared for	707	26.9	63.1	< .001	26.9	43.3	0.03	6.46	< .001	[0.11, 0.21]	0.35

Same-Sex Attractions – Long-Term Relationships											
Reactions	*n*	1–2	3–5	*p*	1–2	4–5	*SE*	*z*	*p*	95% CI	*h*
I would do it	300	58.7	41.3	< .001	58.7	16.0	0.04	− 10.81	< .001	[− 0.50, − 0.36]	0.92
I would consider doing it	300	47.0	53.0	**.142**	47.0	29.3	0.04	− 4.46	< .001	[− 0.25, − 0.10]	0.37
I would find it sexually exciting	300	28.4	71.6	< .001	28.4	46.6	0.04	4.60	< .001	[0.11, 0.26]	0.38
I would be disgusted	300	80.1	19.9	< .001	80.1	7.6	0.04	− 17.89	< .001	[− 0.78, − 0.67]	1.66
I would consider breaking up	300	69.2	30.8	< .001	69.2	14.7	0.04	− 13.53	< .001	[− 0.61, − 0.48]	1.18
I would get angry	300	61.0	39.0	< .001	61.0	21.3	0.04	− 9.88	< .001	[− 0.47, − 0.32]	0.83
I would feel sad	300	59.0	41.0	< .001	59.0	22.3	0.04	− 9.15	< .001	[− 0.44, − 0.29]	0.77
I would feel uncared for	300	55.7	44.3	.005	55.7	26.0	0.04	− 7.40	< .001	[− 0.37, − 0.22]	0.61

Same-Sex Attractions – Short-Term Relationships											
Reaction	*n*	1–2	3–5	*p*	1–2	4–5	*SE*	*z*	*p*	95% CI	*h*
I would do it	300	67.0	33.0	< .001	67.0	14.3	0.04	− 13.14	< .001	[− 0.59, − 0.46]	1.14
I would consider doing it	300	60.0	40.0	< .001	60.0	21.7	0.04	− 9.54	< .001	[− 0.46, − 0.31]	0.80
I would find it sexually exciting	300	31.2	68.8	< .001	31.2	45.6	0.04	3.63	< .001	[0.07, 0.22]	0.30
I would be disgusted	300	83.6	16.4	< .001	83.6	4.4	0.04	− 19.54	< .001	[− 0.84, − 0.74]	1.89
I would consider breaking up	300	55.0	45.0	**.**014	55.0	24.7	0.04	− 7.58	< .001	[− 0.38, − 0.23]	0.63
I would get angry	300	67.0	33.0	< .001	67.0	17.0	0.04	− 12.41	< .001	[− 0.57, − 0.43]	1.07
I would feel sad	300	72.9	27.1	< .001	72.9	11.7	0.04	− 15.17	< .001	[− 0.67, − 0.55]	1.35
I would feel uncared for	300	49.8	50.2	**.922**	49.8	26.8	0.04	− 5.79	< .001	[− 0.31, − 0.15]	0.48

Exclusively Heterosexual – Short-Term Relationships											
Reactions	*n*	1–2	3–5	*p*	1–2	4–5	*SE*	*z*	*p*	95% CI	*h*
I would do it	407	89.5	10.5	< .001	89.5	4.0	0.03	− 24.45	< .001	[− 0.89, − 0.82]	2.08
I would consider doing it	407	85.1	14.9	< .001	85.1	5.0	0.03	− 22.97	< .001	[− 0.84, − 0.76]	1.90
I would find it sexually exciting	407	73.9	26.1	< .001	73.9	11.7	0.03	− 17.93	< .001	[− 0.67, − 0.57]	1.37
I would be disgusted	407	42.5	57.5	< .001	42.5	39.5	0.03	− 0.87	**.384**	[− 0.10, 0.04]	0.06
I would consider breaking up	407	26.7	73.3	< .001	26.7	53.1	0.03	7.69	< .001	[0.20, 0.33]	0.55
I would get angry	407	38.5	61.5	< .001	38.5	41.3	0.03	0.82	**.415**	[− 0.04, 0.10]	0.06
I would feel sad	407	48.8	51.2	**.494**	48.8	30.3	0.03	− 5.40	< .001	[− 0.25, − 0.12]	0.38
I would feel uncared for	407	26.2	73.8	< .001	26.2	52.1	0.03	7.57	< .001	[0.19, 0.32]	0.54

For results concerning all women, the proportions have been weighted (Apostolou & Christoforou, 2018a, pp. 137–138). This table compares affirmative (4–5 as unskewed results, 3–5 as original results) with unaffirmative responses (1–2) from Likert scales. Original results indicate cases where responses 3–5 were considered affirmative, while unskewed results consider only responses 4–5 as affirmative. Percentages may not sum to 100 due to rounding. A two−tailed two−sample proportion *t*−test was employed for all statistical analyses. *h* = Cohen’s *h*, a measure of effect size for the difference between two proportions. Statistical significance was set at *p* < .05 prior to adjustment. Bold text indicates *p* ≥ .05 after Benjamini–Hochberg correction for multiple comparisons

Focusing on heterosexual women with same-sex attractions, in long-term relationships, Apostolou’s original data showed that 71.6% found it sexually exciting, 53.0% would consider it, and 41.3% would do it. However, the unskewed data show that only 46.6% found it sexually exciting (while 28.4% did not, *p* < .001), only 29.3% would consider it (while 47.0% would not, *p* < .001), and only 16.0% would do it (while 58.7% would not, *p* < .001).

In short-term relationships, Apostolou’s original data showed that 68.8% found it sexually exciting, 40.0% would consider it, and 33.0% would do it. However, the unskewed data show that only 45.6% found it sexually exciting (while 31.2% did not, *p* < .001), only 21.7% would consider it (while 60.0% would not, *p* < .001), and only 14.3% would do it (while 67.0% would not, *p* < .001) (Table 1).

Regarding negative reactions in response to their partner’s expressed desire for them to have sex with another woman, in long-term relationships, Apostolou’s original data showed that, for example, 55.8% would get angry, and 59.9% would feel sad. However, the unskewed results show that only 39.4% would get angry (while 34.2% would not, *p* = .043), and only 42.9% would feel sad (while 30.1% would not, *p* < .001).

In short-term relationships, Apostolou’s original data showed that, for example, 62.0% would consider breaking up, and 63.1% would feel uncared for by their partner. However, the unskewed results show that only 43.8% would consider breaking up (while 28.0% would not, *p* < .001), and only 43.3% would feel uncared for by their partner (while 26.9% would not, *p* < .001) (Table 1).

#### Same-Sex Contact Involving Partner Participation

In response to their partner’s expressed desire for them to have sex with another woman while their partner participates, in long-term relationships, Apostolou’s original data showed that 27.6% found it sexually exciting, 24.2% would consider it, and 19.3% would do it. However, the unskewed data show that only 15.5% found it sexually exciting (while 62.4% did not, *p* < .001), only 11.4% would consider it (while 65.8% would not, *p* < .001), and only 8.4% would do it (while 70.7% would not, *p* < .001). Once again, the unskewed data contradict Apostolou’s predictions.

In short-term relationships, Apostolou’s original data showed that 32.1% found it sexually exciting, 22.2% would consider it, and 20.7% would do it. However, the unskewed data show that only 18.5% found it sexually exciting (while 57.9% did not, *p* < .001), only 10.3% would consider it (while 67.8% would not, *p* < .001), and only 7.7% would do it (while 70.7% would not, *p* < .001) (Table 2). A two-sample proportion *t*-test based on the unskewed results revealed no significant differences between the long-term and short-term contexts in the propensity to find it sexually exciting (*z* = 1.50, *p* = .133), to consider it (*z* = 0.67, *p* = .506), or to do it (*z* = 0.70, *p* = .486).

**Table 2:** Women’s preferences for same-sex sexual contact and cheating

Same-Sex Sexual Contact With Partner Participation (Apostolou & Christoforou, 2018a)											
All Women – Long-Term Relationships											
		Original results (%)			Unskewed results (%)						
Behavior	*N*	1–2	3–5	*p*	1–2	4–5	*SE*	*z*	*p*	95% CI	*h*
I would do it	707	70.7	19.3	< .001	70.7	8.4	0.03	− 23.96	< .001	[− 0.66, − 0.58]	1.41
I would consider doing it	707	65.8	24.2	< .001	65.8	11.4	0.03	− 21.01	< .001	[− 0.59, − 0.50]	1.20
I would find it sexually exciting	707	62.4	27.6	< .001	62.4	15.5	0.03	− 18.08	< .001	[− 0.51, − 0.42]	1.01
I would be disgusted	707	37.7	52.2	< .001	37.7	36.1	0.03	− 0.62	**.533**	[− 0.07, 0.03]	0.03
I would consider breaking up	707	35.7	54.3	< .001	35.7	40.1	0.03	1.71	**.088**	[− 0.007, 0.10]	0.09
I would get angry	707	26.1	63.9	< .001	26.1	50.1	0.03	9.29	< .001	[0.19, 0.29]	0.50
I would feel sad	707	26.2	63.8	< .001	26.2	50.1	0.03	9.25	< .001	[0.19, 0.29]	0.50
I would feel uncared for	707	28.5	61.5	< .001	28.5	44.5	0.03	6.25	< .001	[0.11, 0.21]	0.33

All Women – Short-Term Relationships											
Behavior	*N*	1–2	3–5	*p*	1–2	4–5	*SE*	*z*	*p*	95% CI	*h*
I would do it	707	70.7	20.7	< .001	70.7	7.7	0.03	− 24.26	< .001	[− 0.67, − 0.59]	1.44
I would consider doing it	707	67.8	22.2	< .001	67.8	10.3	0.03	− 22.16	< .001	[− 0.62, − 0.53]	1.28
I would find it sexually exciting	707	57.9	32.1	< .001	57.9	18.5	0.03	− 15.25	< .001	[− 0.44, − 0.35]	0.84
I would be disgusted	707	39.8	50.1	< .001	39.8	35.7	0.03	− 1.59	**.112**	[− 0.09, 0.009]	0.09
I would consider breaking up	707	28.6	61.4	< .001	28.6	44.6	0.03	6.24	< .001	[0.11, 0.21]	0.33
I would get angry	707	35.9	54.1	< .001	35.9	34.0	0.03	− 0.75	**.454**	[− 0.07, 0.03]	0.04
I would feel sad	707	44.6	45.4	**.762**	44.6	25.4	0.03	− 7.57	< .001	[− 0.24, − 0.14]	0.41
I would feel uncared for	707	27.5	62.5	< .001	27.5	44.5	0.03	6.66	< .001	[0.12, 0.22]	0.36

Same-Sex Attractions – Long-Term Relationships											
Behavior	*n*	1–2	3–5	*p*	1–2	4–5	*SE*	*z*	*p*	95% CI	*h*
I would do it	300	59.3	40.7	< .001	59.3	18.0	0.04	− 10.39	< .001	[− 0.48, − 0.34]	0.88
I would consider doing it	300	50.6	49.4	**.769**	50.6	28.7	0.04	− 5.48	< .001	[− 0.30, − 0.14]	0.45
I would find it sexually exciting	300	38.1	61.9	< .001	38.1	38.6	0.04	0.13	**.900**	[− 0.07, 0.08]	0.01
I would be disgusted	300	73.9	26.1	< .001	73.9	14.4	0.04	− 14.68	< .001	[− 0.66, − 0.53]	1.29
I would consider breaking up	300	60.9	39.1	< .001	60.9	19.4	0.04	− 10.37	< .001	[− 0.49, − 0.34]	0.88
I would get angry	300	54.3	45.7	.035	54.3	31.4	0.04	− 5.67	< .001	[− 0.31, − 0.15]	0.47
I would feel sad	300	49.5	50.5	**.806**	49.5	31.8	0.04	− 4.41	< .001	[− 0.25, − 0.10]	0.36
I would feel uncared for	300	54.6	45.4	.024	54.6	30.4	0.04	− 6.00	< .001	[− 0.32, − 0.17]	0.49

Same-Sex Attractions – Short-Term Relationships											
Behavior	*n*	1–2	3–5	*p*	1–2	4–5	*SE*	*z*	*p*	95% CI	*h*
I would do it	300	56.3	43.7	.002	56.3	20.0	0.04	− 9.15	< .001	[− 0.44, − 0.29]	0.77
I would consider doing it	300	53.0	47.0	**.142**	53.0	24.9	0.04	− 7.06	< .001	[− 0.36, − 0.21]	0.59
I would find it sexually exciting	300	30.1	69.9	< .001	30.1	46.8	0.04	4.20	< .001	[0.09, 0.24]	0.35
I would be disgusted	300	80.9	19.1	< .001	80.9	10.1	0.04	− 17.41	< .001	[− 0.76, − 0.65]	1.59
I would consider breaking up	300	58.4	41.6	< .001	58.4	20.8	0.04	− 9.42	< .001	[− 0.45, − 0.30]	0.79
I would get angry	300	67.2	32.8	< .001	67.2	15.7	0.04	− 12.80	< .001	[− 0.58, − 0.45]	1.11
I would feel sad	300	72.9	27.1	< .001	72.9	13.2	0.04	− 14.77	< .001	[− 0.66, − 0.53]	1.30
I would feel uncared for	300	53.1	46.9	**.129**	53.1	26.1	0.04	− 6.76	< .001	[− 0.35, − 0.19]	0.56

Exclusively Heterosexual – Long-Term Relationships											
Behavior	*n*	1–2	3–5	*p*	1–2	4–5	*SE*	*z*	*p*	95% CI	*h*
I would do it	407	82.1	17.9	< .001	82.1	7.7	0.03	− 21.34	< .001	[− 0.79, − 0.70]	1.71
I would consider doing it	407	77.2	22.8	< .001	77.2	9.7	0.03	− 19.43	< .001	[− 0.72, − 0.63]	1.51
I would find it sexually exciting	407	75.1	24.9	< .001	75.1	13.3	0.03	− 17.75	< .001	[− 0.67, − 0.56]	1.35
I would be disgusted	407	36.0	64.0	< .001	36.0	44.9	0.03	2.59	.010	[0.02, 0.16]	0.18
I would consider breaking up	407	35.7	64.3	< .001	35.7	49.2	0.03	3.90	< .001	[0.07, 0.20]	0.27
I would get angry	407	24.3	75.7	< .001	24.3	60.2	0.03	10.37	< .001	[0.30, 0.42]	0.75
I would feel sad	407	25.3	74.7	< .001	25.3	60.1	0.03	10.04	< .001	[0.28, 0.41]	0.72
I would feel uncared for	407	27.4	72.6	< .001	27.4	53.9	0.03	7.70	< .001	[0.20, 0.33]	0.55

Exclusively Heterosexual – Short-Term Relationships											
Behavior	*n*	1–2	3–5	*p*	1–2	4–5	*SE*	*z*	*p*	95% CI	*h*
I would do it	407	82.7	17.3	< .001	82.7	6.4	0.03	− 21.90	< .001	[− 0.81, − 0.72]	1.77
I would consider doing it	407	79.5	20.5	< .001	79.5	8.9	0.03	− 20.28	< .001	[− 0.75, − 0.66]	1.60
I would find it sexually exciting	407	70.6	29.4	< .001	70.6	15.7	0.03	− 15.81	< .001	[− 0.61, − 0.49]	1.18
I would be disgusted	407	37.5	62.5	< .001	37.5	45.1	0.03	2.20	.028	[0.009, 0.14]	0.15
I would consider breaking up	407	26.9	73.1	< .001	26.9	54.9	0.03	8.12	< .001	[0.22, 0.34]	0.58
I would get angry	407	34.9	65.1	< .001	34.9	41.8	0.03	2.02	.043	[0.002, 0.14]	0.14
I would feel sad	407	45.3	54.7	.007	45.3	31.0	0.03	− 4.20	< .001	[− 0.21, − 0.08]	0.30
I would feel uncared for	407	26.4	73.6	< .001	26.4	53.8	0.03	7.98	< .001	[0.21, 0.34]	0.57

Cheating Preferences											
All Women – Long-Term Relationships											
		Original results (%)			Unskewed results (%)						
Behavior	*N*	1–2	3–5	*p*	1–2	4–5	*SE*	*z*	*p*	95% CI	*h*
I would have sex with a man	707	49.9	40.1	< .001	49.9	25.6	0.03	− 9.42	< .001	[− 0.29, − 0.19]	0.51
I would have sex with a woman	707	78.6	11.4	< .001	78.6	4.6	0.03	− 28.23	< .001	[− 0.77, − 0.71]	1.75

All Women – Short-Term Relationships											
I would have sex with a man	707	26.2	63.8	< .001	26.2	48.1	0.03	8.52	< .001	[0.17, 0.27]	0.46
I would have sex with a woman	707	71.9	18.1	< .001	71.9	9.0	0.03	− 24.10	< .001	[− 0.67, − 0.59]	1.41

Same-Sex Attractions – Long-Term Relationships											
Behavior	*n*	1–2	3–5	*p*	1–2	4–5	*SE*	*z*	*p*	95% CI	*h*
I would have sex with a man	300	52.7	47.3	**.186**	52.7	30.6	0.04	− 5.49	< .001	[− 0.30, − 0.14]	0.45
I would have sex with a woman	300	56.8	43.2	< .001	56.8	24.8	0.04	− 7.97	< .001	[− 0.39, − 0.25]	0.66

Same-Sex Attractions – Short-Term Relationships											
I would have sex with a man	300	27.1	72.9	< .001	27.1	58.2	0.04	7.70	< .001	[0.24, 0.39]	0.64
I would have sex with a woman	300	37.9	62.1	< .001	37.9	42.3	0.04	1.10	**.272**	[− 0.03, 0.12]	0.09

Exclusively Heterosexual – Long-Term Relationships											
Behavior	*n*	1–2	3–5	*p*	1–2	4–5	*SE*	*z*	*p*	95% CI	*h*
I would have sex with a man	407	56.0	44.0	< .001	56.0	28.0	0.03	− 8.09	< .001	[− 0.35, − 0.21]	0.58
I would have sex with a woman	407	93.0	7.0	< .001	93.0	1.5	0.03	− 26.15	< .001	[− 0.94, − 0.89]	2.36

Exclusively Heterosexual – Short-Term Relationships											
I would have sex with a man	407	29.5	70.5	< .001	29.5	52.6	0.03	6.70	< .001	[0.17, 0.30]	0.47
I would have sex with a woman	407	87.6	12.4	< .001	87.6	4.1	0.03	− 23.91	< .001	[− 0.87, − 0.80]	2.01

For results concerning all women, the proportions have been weighted (Apostolou & Christoforou, 2018a, pp. 137–138). This table compares affirmative (4–5 as unskewed results, 3–5 as original results) with unaffirmative responses (1–2) from Likert scales. Original results indicate cases where responses 3–5 were considered affirmative, while unskewed results consider only responses 4–5 as affirmative. Percentages may not sum to 100 due to rounding. A two−tailed two−sample proportion *t*−test was employed for all statistical analyses. *h* = Cohen’s *h*, a measure of effect size for the difference between two proportions. Statistical significance was set at *p* < .05 prior to adjustment. Bold text indicates *p* ≥ .05 after Benjamini–Hochberg correction for multiple comparisons

Focusing on women with same-sex attractions, in long-term relationships, Apostolou’s original data showed that 61.9% found it sexually exciting, 49.4% would consider it, and 40.7% would do it. However, the unskewed data show that only 38.6% found it sexually exciting (while 38.1% did not, *p* = .900), only 28.7% would consider it (while 50.6% would not, *p* < .001), and only 18.0% would do it (while 59.3% would not, *p* < .001).

In short-term relationships, Apostolou’s original data showed that 69.9% found it sexually exciting, 47.0% would consider it, and 43.7% would do it. However, the unskewed data show that only 46.8% found it sexually exciting (while 30.1% did not, *p* < .001), only 24.9% would consider it (while 53.0% would not, *p* < .001), and only 20.0% would do it (while 56.3% would not, *p* < .001) (Table 2). A two-sample proportion *t*-test based on the unskewed results revealed no significant differences between the long-term and short-term contexts in the propensity to consider it (*z* = 1.05, *p* = .293) or to do it (*z* = 0.62, *p* = .532).

Among exclusively heterosexual women, a two-sample proportion *t*-test based on the unskewed results revealed no significant differences between the long-term and short-term contexts in the propensity to find it sexually exciting (*z* = 0.97, *p* = .331), to consider it (*z* = 0.39, *p* = .694), or to do it (*z* = 0.72, *p* = .469).

Regarding negative reactions in response to their partner’s expressed desire for them to have sex with another woman while their partner participates, in long-term relationships, Apostolou’s original data showed that, for example, 63.9% would get angry, 63.8% would feel sad, and 61.5% would feel uncared for by their partner. However, the unskewed data show that only 50.1% would get angry (while 26.1% would not, *p* < .001), only 50.1% would feel sad (while 26.2% would not, *p* < .001), and only 44.5% would feel uncared for by their partner (while 28.5% would not, *p* < .001).

In short-term relationships, Apostolou’s original data showed that, for example, 61.4% would consider breaking up, and 62.5% would feel uncared for by their partner. However, the unskewed data show that only 44.6% would consider breaking up (while 28.6% would not, *p* < .001), and only 44.5% would feel uncared for by their partner (while 27.5% would not, *p* < .001) (Table 2).

#### Cheating Preferences

In a scenario where women considered cheating, in long-term relationships, Apostolou’s original data showed that 11.4% would likely cheat with another woman, and 40.0% would likely cheat with a man. However, the unskewed data show that only 4.6% would likely cheat with another woman, and only 25.6% would likely cheat with a man (*p* < .001).

In short-term relationships, Apostolou’s original data showed that 18.1% would likely cheat with another woman, and 63.8% would likely cheat with a man. However, the unskewed data show that only 9.0% would likely cheat with another woman, and only 48.1% would likely cheat with a man (*p* < .001) (Table 2). A two-sample proportion *t*-test based on the unskewed results revealed that women were more likely to cheat with another woman in a short-term compared to a long-term context (*z* = 3.29, *p* = .001), but at the same time, the propensity to cheat with a man rather than a woman was *greater* in a short-term than a long-term context (Cohen’s *h* = 0.92 vs. 0.63; *h* measures effect size, showing a larger difference in the short-term than in the long-term context). The same phenomenon was observed among women with same-sex attractions (Cohen’s *h* = 0.32 vs. 0.13).

### Findings on Men’s Responses to a Partner’s Same-Sex Sexual Infidelity

Three studies of Greek, Greek-Cypriot, Chinese, and British samples examined heterosexual men’s reactions to an imagined scenario of their partner’s same-sex infidelity (Apostolou, 2019b; Wang & Apostolou, 2019). Unskewed results were extracted, and weighted percentages with standard deviations were calculated to account for differing sample sizes.

In long-term relationships, Apostolou’s original data showed that 76.3% (*SD* = 8.2%) would consider an act of same-sex contact by their partner as cheating, 70.9% (*SD* = 4.9%) would consider breaking up, 76.1% (*SD* = 4.4%) would get angry, and 73.3% (*SD* = 6.5%) would become very sad. However, the unskewed data show that only 59.7% (*SD* = 6.5%) would consider an act of same-sex contact by their partner as cheating (while 23.7% [*SD* = 8.2%] would not, *p* < .001), only 47.4% (*SD* = 2.1%) would consider breaking up (while 29.1% [*SD* = 4.9%] would not, *p* < .001), only 60.2% (*SD* = 5.6%) would get angry (while 23.9% [*SD* = 4.4%] would not, *p* < .001), and only 54.4% (*SD* = 3.4%) would become very sad (while 26.8% [*SD* = 6.5%] would not, *p* < .001).

In short-term relationships, Apostolou’s original data showed that 62.8% (*SD* = 10.3%) would consider an act of same-sex contact by their partner as cheating. However, the unskewed data show that only 43.1% (*SD* = 9.4%) would consider an act of same-sex contact by their partner as cheating (while 37.2% [*SD* = 10.5%] would not, *p* = .019) (Table 3).

**Table 3:** Men’s reactions and sexual excitement in response to a partner’s same-sex sexual infidelity

Reactions to Partner’s Same-Sex Sexual Infidelity (Apostolou, 2019b; Wang & Apostolou, 2019)											
Long-Term Relationships											
		Original results % (*SD*)			Unskewed results % (*SD*)						
Behavior	*N*	1–2	3–5	*p*	1–2	4–5	*SE*	*z*	*p*	95% CI	*h*
I would consider that she has cheated	759	23.7 (8.2)	76.3 (10.1)	< .001	23.7 (8.2)	59.7 (6.5)	0.03	14.22	< .001	[0.31, 0.41]	0.75
I would break up	759	29.1 (4.9)	70.9 (5.6)	< .001	29.1 (4.9)	47.4 (2.1)	0.02	7.34	< .001	[0.13, 0.23]	0.38
I would get angry	759	23.9 (4.4)	76.1 (5.4)	< .001	23.9 (4.4)	60.2 (5.6)	0.03	14.33	< .001	[0.32, 0.41]	0.75
I would get jealous	759	43.9 (9.8)	56.1 (12.0)	< .001	43.9 (9.8)	40.0 (10.0)	0.03	− 1.54	**.124**	[− 0.09, 0.01]	0.08
I would become very sad	759	26.8 (6.5)	73.3 (7.9)	< .001	26.8 (6.5)	54.4 (3.4)	0.03	10.95	< .001	[0.23, 0.32]	0.57

Short-Term Relationships											
Behavior	*N*	1–2	3–5	*p*	1–2	4–5	*SE*	*z*	*p*	95% CI	*h*
I would consider that she has cheated	759	37.2 (10.5)	62.8 (12.6)	< .001	37.2 (10.5)	43.1 (9.4)	0.03	2.34	.019	[0.01, 0.11]	0.12
I would break up	759	37.4 (6.9)	62.6 (8.4)	< .001	37.4 (6.9)	39.7 (5.3)	0.02	0.92	**.357**	[− 0.03, 0.07]	0.05
I would get angry	759	37.4 (10.6)	62.6 (13.0)	< .001	37.4 (10.6)	41.7 (12.2)	0.03	1.71	**.087**	[− 0.006, 0.09]	0.09
I would get jealous	759	49.7 (9.2)	50.3 (11.3)	**.815**	49.7 (9.2)	31.7 (9.7)	0.03	− 7.14	< .001	[− 0.23, − 0.13]	0.37
I would become very sad	759	43.7 (9.2)	56.3 (11.3)	< .001	43.7 (9.2)	35.1 (10.4)	0.03	− 3.43	< .001	[− 0.14, − 0.04]	0.18

Reactions to Partner’s Same-Sex Sexual Infidelity – Sexual Excitement (Apostolou et al., 2017, 2018)											
Long-Term Relationships											
Behavior	*N*	1–2	3–5	*p*	1–2	4–5	*SE*	*z*	*p*	95% CI	*h*
I would get sexually excited	759	60.6 (9.1)	49.2 (13.4)	< .001	60.6 (9.1)	25.4 (5.6)	0.03	− 13.85	< .001	[− 0.40, − 0.31]	0.73

Short-Term Relationships											
I would get sexually excited	759	51.7 (10.5)	59.6 (2.3)	.002	51.7 (10.5)	32.6 (9.3)	0.03	− 7.54	< .001	[− 0.24, − 0.14]	0.39

This table compares affirmative (4–5 as unskewed results, 3–5 as original results) with unaffirmative responses (1–2) from Likert scales. Original results indicate cases where responses 3–5 were considered affirmative, while unskewed results consider only responses 4–5 as affirmative. Percentages may not sum to 100 due to rounding. A two−tailed two−sample proportion *t*−test was employed for all statistical analyses. *h* = Cohen’s *h*, a measure of effect size for the difference between two proportions. Statistical significance was set at *p* < .05 prior to adjustment. Bold text indicates *p* ≥ .05 after Benjamini–Hochberg correction for multiple comparisons

Three studies of Greek, Greek-Cypriot, Chinese, and British samples examined heterosexual men’s sexual excitement in response to an imagined scenario of their partner’s same-sex infidelity (Apostolou et al., 2017, 2018).^2^ Unskewed results were extracted, and weighted percentages with standard deviations were calculated to account for differing sample sizes.

In long-term relationships, Apostolou’s original data showed that 49.2% (*SD* = 13.4%) found it sexually exciting. However, the unskewed data show that only 25.4% (*SD* = 5.6%) found it sexually exciting (while 60.6% [*SD* = 9.1%] did not, *p* < .001).

In short-term relationships, Apostolou’s original data showed that 59.6% (*SD* = 2.3%) found it sexually exciting. However, the unskewed data show that only 32.6% (*SD* = 9.3%) found it sexually exciting (while 51.7% [*SD* = 10.5%] did not, *p* < .001) (Table 3). Once again, the unskewed data show significant differences in the opposite direction to what Apostolou predicted.

## Discussion and Interpretation of Results

### Evaluation of the Cuckoldry-Protection Mechanism

The cuckoldry protection mechanism claims that heterosexual women engage in same-sex activity to safeguard their partner’s paternity. Yet, the unskewed data do not support this claim: Only 1 in 19 women would engage in such behavior, only 1 in 10 would consider it, and only 1 in 7 find it sexually exciting. Negative reactions were common, with many women in short-term relationships even contemplating breakups. When asked if they would cheat with someone of either sex, women overwhelmingly chose men— regardless of whether they had same-sex attractions or whether the context was short- or long-term—contradicting a cuckoldry protection mechanism. Moreover, given the very low prevalence of same-sex interest, it is improbable that this behavior could serve as an effective cuckoldry-mitigation strategy. If same-sex attraction served as cuckoldry protection, men would likely prefer partners with this trait, similar to preferences for chastity and fidelity (Buss, 1989; Buss & Schmitt, 1993). However, most men do not (see Supplement Material), and many respond negatively to same-sex infidelity, often considering breakups. The lack of female interest in same-sex contact, combined with men’s disinterest in partners with same-sex attractions, suggests that this trait may not function as an evolved cuckoldry protection mechanism.

### Evaluation of the "Participation Effect" Mechanism

The "participation effect" mechanism claims that men prefer female partners with same-sex attractions for shared sexual encounters. Yet, the unskewed data do not support this claim: If such an effect existed, women would exhibit strong same-sex inclinations, and men would prefer such partners, especially in short-term relationships. However, women generally show little interest in same-sex contact, and most men in long-term relationships do not desire it, even when it allows them to participate sexually (see Supplement Material). While men in short-term relationships are more open, they still view same-sex contact by their partner as cheating. Given men’s preference for sexual variety (Schmitt et al., 2003; Symons, 1979) and pursuit of casual mating (Clark & Hatfield, 1989; Hald & Høgh-Olesen, 2010), they would likely prefer women with same-sex attractions if it enhanced reproductive opportunities. However, women’s disinterest in same-sex contact, coupled with men’s lack of preference for it, suggests these attractions do not increase male reproductive fitness through increased mating opportunities.

### Evaluation of the Enhanced Relationship Quality Mechanism

The enhanced relationship quality mechanism claims that same-sex attractions provide fitness benefits through enhanced relationship dynamics. Yet, the unskewed data do not support this claim: If such benefits existed, women with same-sex attractions would likely be more open to same-sex encounters suggested by their partners. However, only 1 in 7 to 1 in 5 women would consider it, and only 1 in 5 to 1 in 3 would entertain the idea. Similarly, the notion that same-sex attractions help manage a partner’s extra-pair mating by redirecting it to another woman is unsupported. Only 1 in 5 women would consider sex with another woman if their partner could participate, and only 1 in 4 would entertain this scenario. Moreover, heterosexual men do not prefer partners with same-sex attractions (see Supplement Material). In conclusion, there is no evidence that same-sex attractions in heterosexual women provide fitness benefits through enhanced relationship dynamics.

Across the analyses underlying the evaluation of the three proposed mechanisms, the observed patterns are consistent and robust to alternative treatments of midpoint responses. Even if midpoint responses were included rather than treated as analytically indeterminate, it is highly improbable that the overall conclusions would change. Because the present reanalysis relied on the percentage distributions reported in the original publications rather than raw datasets, future studies using newly collected datasets would provide a more definitive test of the male choice hypothesis.

### Alternative Evolutionary Hypotheses

Given the shortcomings of the male choice hypothesis, alternative evolutionary explanations deserve consideration. Two evolutionary psychology theories provide more plausible adaptive accounts of same-sex attractions in heterosexual women: the alloparenting hypothesis (Kuhle & Radtke, 2013) and the polygyny hypothesis (Kanazawa, 2017). Both link female sexual fluidity to reproductive success, but via different mechanisms: the alloparenting hypothesis proposes it evolved as a conditional strategy to secure allomothering when paternal investment was absent, whereas the polygyny hypothesis conceptualizes it as reducing conflict among co-wives, facilitating cooperation, and enhancing child-rearing. Both account for sexual fluidity, but the polygyny hypothesis is more comprehensive in scope and consistent with broader empirical patterns, including women’s greater erotic plasticity, weaker category-specific sexual arousal, and heterosexual men’s widespread arousal to female–female sex, a pattern documented cross-culturally (Rowland & Uribe, 2020). Under the alloparenting hypothesis, any male fitness benefits are incidental and not posited as drivers of selection for male arousal. In contrast, the polygyny hypothesis treats male arousal as functionally relevant, as it would be expected to promote co-wife harmony, stabilize polygynous groups, and enhance men’s reproductive success.

### Conclusions

Apostolou’s male choice hypothesis seeks to explain the adaptive value of same-sex attractions in heterosexual women. However, the hypothesis does not provide a satisfactory evolutionary account. It exhibits core theoretical limitations that are difficult to reconcile with established evolutionary principles and conflicts with well-supported empirical findings. In addition, a reanalysis of Apostolou et al.’s own data indicates that their results do not support the hypothesis. Given these substantial shortcomings, the male choice hypothesis exhibits significant theoretical and empirical limitations. Finally, alternative evolutionary hypotheses offer more plausible and empirically grounded explanations for same-sex attractions among heterosexual women.

## Supplementary Information

Below is the link to the electronic supplementary material.

Supplementary data (DOCX 26kb docx)

## Data Availability

The data analyzed in this article were extracted from published tables in previously conducted studies. Detailed references to these studies are provided in the main text.

## Footnotes

1 In the original studies, McNemar’s test was used due to access to raw data, allowing for paired responses. In this review, only aggregated proportions are available, making McNemar’s test unsuitable due to potential assumption violations. Instead, a two-proportion *t*-test is used, assuming independent samples and providing valid comparisons between long-term and short-term conditions. This method is applied throughout for all such comparisons.

2 Another study by Apostolou (2016b) (*n* = 220) also examined heterosexual men’s sexual excitement in response to an imagined scenario of their partner’s same-sex infidelity but was not included in this reanalysis due to the skewed reporting of responses, which prevented accurate extraction of unskewed results.
