# Supplementary material for: Male Choice Hypothesis: Shortcomings, Inconsistencies, and Proposed Alternatives
Source: Arch Sex Behav. 2026 Jul 13;55(5):2071–86. doi: 10.1007/s10508-026-03469-3 (PMC13427796; doi:10.1007/s10508-026-03469-3)
Supplement: Supplementary file 1 — Supplementary data (DOCX 26kb docx) [file 10508_2026_3469_MOESM1_ESM.docx]

**Supplemental Material**

**Men's Preferences for Same-Sex Attractions in a Partner**

Five studies of Greek-Cypriot, Chinese, and British samples examined heterosexual men's preferences for partners with same-sex attractions and desires for their partners to have same-sex contact (Apostolou, 2019a; Apostolou & Christoforou, 2018b; Apostolou et al., 2017, 2018). Weighted percentages with standard deviations were calculated to account for differing sample sizes.

***Preference for Same-Sex Attractions and Behaviors in a Partner***

In long-term relationships, 73.3% (*SD* = 11.7%) preferred a partner attracted only to men, while 26.7% (*SD* = 11.6%) preferred a partner attracted to some degree toward women (*p* < .001). Seventy-nine point four percent (*SD* = 5.3%) preferred their partner to never engage sexually with other women, while 20.6% (*SD* = 5.3%) preferred their partner to engage sexually with other women occasionally or frequently (*p* < .001) (Table S1).

In short-term relationships, 56.2% (*SD* = 15.1%) preferred a partner attracted only to men, while 43.9% (*SD* = 15.2%) preferred a partner attracted to some degree toward women (*p* < .001). Sixty-two point two percent (*SD* = 10.5%) preferred their partner to never engage sexually with other women, while 37.8% (*SD* = 10.6%) preferred their partner to engage sexually with other women occasionally or frequently (*p* < .001) (Table S1).

***Participation in a Partner's Same-Sex Sexual Contact***

In the scenario of their partner having sexual contact with other women where the men would also participate, in long-term relationships, 55.9% (*SD* = 5.9%) preferred their partner to never engage sexually with other women, while 44.0% (*SD* = 4.2%) preferred their partner to engage sexually with other women occasionally or frequently (*p* < .001). In short-term relationships, 39.2% (*SD* = 3.2%) preferred their partner to never engage sexually with other women, while 60.9% (*SD* = 4.5%) preferred their partner to engage sexually with other women occasionally or frequently (*p* < .001) (Table S1).

**Table S1**

*Men's Preferences for Partner's Same-Sex Attractions, Behavior, and Participation in Same-Sex Contact*

| Preference for Same-Sex Attractions in Partner (Apostolou 2019a; Apostolou & Christoforou 2018b; Apostolou et al., 2018). *N* = 1,981 | | | | | | | | | |
| --- | --- | --- | --- | --- | --- | --- | --- | --- | --- |
|  | | | | Long-term |  |  | Short-term |  |  |
| I prefer a partner attracted | | | | Prevalence (%) | *SD* | *p* | Prevalence (%) | *SD* | *p* |
| Only to the opposite sex | | | | 73.3 | 11.7 | < .001 | 56.2 | 15.1 | < .001 |
| Predominantly to the opposite sex | | | | 19.5 | 8.3 |  | 26.3 | 8.3 |  |
| To both sexes equally | | | | 3.7 | 3.2 |  | 13.0 | 8.0 |  |
| Predominantly to the same sex | | | | 1.3 | 0.7 |  | 1.9 | 0.4 |  |
| Only to the same sex | | | | 2.1 | 1.7 |  | 2.7 | 1.8 |  |
|  | | | |  |  |  |  |  |  |
| Preference for Same-Sex Behavior in Partner (Apostolou, 2019a; Apostolou & Christoforou, 2018b; Apostolou et al. (2017, 2018). *N* = 2,222 | | | | | | | | | |
|  | | | | Long-term |  |  | Short-term |  |  |
| I prefer my partner has same-sex sexual contact | | | | Prevalence (%) | *SD* | *p* | Prevalence (%) | *SD* | *p* |
| Never |  |  |  | 79.4 | 5.3 | < .001 | 62.2 | 10.5 | < .001 |
| Occasionally |  |  |  | 18.2 | 5.5 |  | 29.3 | 6.7 |  |
| Frequently |  |  |  | 2.4 | 1.0 |  | 8.5 | 4.0 |  |
|  | | | |  |  |  |  |  |  |
| Participation in Partner's Same-Sex Sexual Contact (Apostolou, 2019a; Apostolou & Christoforou, 2018b). *N* = 1,091 | | | | | | | | | |
|  | | | | Long-term |  |  | Short-term |  |  |
| I prefer my partner has same-sex contact in which I can participate | | | | Prevalence (%) | *SD* | *p* | Prevalence (%) | *SD* | *p* |
| Never |  |  |  | 55.9 | 5.9 | < .001 | 39.2 | 3.2 | < .001 |
| Occasionally |  |  |  | 32.5 | 2.3 |  | 39.3 | 0.5 |  |
| Frequently |  |  |  | 11.6 | 3.5 |  | 21.6 | 3.7 |  |

*Note*. This table shows significance levels for comparisons between the first category in each panel (e.g., "Only to the opposite sex," "Never") and the other options within the same panel. Percentages may not sum to 100 due to rounding. A two-tailed two-sample proportion *t*-test was employed for all statistical analyses. Statistical significance was set at *p* < .05 prior to adjustment. Bold text indicates *p* ≥ .05 after Benjamini-Hochberg correction for multiple comparisons.

**References**

Apostolou, M. (2019a). Men's preference for women who like women: The effects of desire for sexual variety and willingness to have sex without commitment. *Evolutionary Psychology*, *17*(3). <https://doi.org/10.1177/1474704919856800>

Apostolou, M., & Christoforou, C. (2018b). Same-sex attraction and contact in an opposite sex partner: Exploring sex, religiosity, porn consumption and participation effects. Personality and Individual Differences, 131, 26–30. [https://doi.org/10.1016/j.paid.2018.04.017](https://psycnet.apa.org/doi/10.1016/j.paid.2018.04.017)

Apostolou, M., Shialos, M., Khalil, M., & Paschali, V. (2017). The evolution of female same-sex attraction: The male choice hypothesis. Personality and Individual Differences, 116, 372–378. <https://doi.org/10.1016/j.paid.2017.05.020>

Apostolou, M., Wang, Y., & O, J. (2018). Do men prefer women who are attracted to women? A cross-cultural evolutionary investigation. *Personality and Individual Differences, 135,* 31–39. [https://doi.org/10.1016/j.paid.2018.06.052](https://psycnet.apa.org/doi/10.1016/j.paid.2018.06.052)
